# Anti-invasive effects of *Celastrus Orbiculatus* extract on interleukin-1 beta and tumour necrosis factor-alpha combination-stimulated fibroblast-like synoviocytes

**DOI:** 10.1186/1472-6882-14-62

**Published:** 2014-02-19

**Authors:** Guoqing Li, Dan Liu, Shiyu Guo, Masataka Sunagawa, Tadashi Hisamitsu, Yanqing Liu

**Affiliations:** 1 Department of Rheumatology, Clinical Medical College, Yangzhou University, Yangzhou 225000, China; 2 Department of Pathology, Clinical Medical College, Yangzhou University, Yangzhou 225000, China; 3 Department of Physiology, School of Medicine, Showa University, Tokyo 142-8555, Japan; 4 Institution of Traditional Chinese Medicine and Western Medicine, Medical College, Yangzhou University, Yangzhou 225000, China

**Keywords:** Celastrus orbiculatus, Invasion, Fibroblast-like synoviocyte, MMP, Rheumatoid arthritis

## Abstract

**Background:**

Invasion of fibroblast-like synoviocytes (FLSs) is critical in the pathogenesis of rheumatoid arthritis (RA). The metalloproteinases (MMPs) and activator of nuclear factor-kappa B (NF-κB) pathway play a critical role in RA-FLS invasion induced by interleukin-1 beta (IL-1β) and tumour necrosis factor-alpha (TNF-α). The present study aimed to explore the anti-invasive activity and mechanism of *Celastrus orbiculatus* extract (COE) on IL-1β and TNF-α combination-stimulated human RA-FLSs.

**Methods:**

We investigated the effect of COE on IL-1β and TNF-α combination-induced FLS invasion as well as MMP expression and explored upstream signal transduction.

**Results:**

COE suppressed IL-1β and TNF-α combination-stimulated FLSs invasion by inhibiting MMP-9 expression and activity. Furthermore, our results revealed that COE inhibited the transcriptional activity of MMP-9 by suppression of the binding activity of NF-κB in the MMP-9 promoter, and inhibited IκBα phosphorylation and nuclear translocation of NF-κB.

**Conclusions:**

COE inhibits IL-1β and TNF-α combination-induced FLSs invasion by suppressing NF-κB-mediated MMP-9 expression.

## Background

Rheumatoid arthritis (RA) is a complex chronic autoimmune disease mainly affecting the joints, characterized by abnormal synovial hyperplasia with marked pannus formation and subsequent invasion and destruction of cartilage and bone [1,2]. Growing evidence suggests that fibroblast-like synoviocytes (FLSs) in the lining layer can attach to the cartilage and invade the extracellular matrix. This aggressive invasive behaviour has an important role in initiating and driving RA [3,4]. The migration of activated FLS is also partly responsible for spreading arthritis destruction to distant joints [5]. FLSs have inherent invasive qualities not observed in other fibroblasts. The invasion of FLSs in RA is considered to be as aggressive as tumor cells [6]. Therefore, the regulation of cell migration and invasion is a critical process throughout the development of RA.

In RA, there is a link between inflammation and increased bone damage. It is well established that pro-inflammatory cytokines are key mediators of RA-FLS invasion and are involved in the pathogenesis of RA [3]. Cytokines, such as interleukin-1β (IL-1β), IL-6 and tumor necrosis factor (TNF-α), can stimulate RA-FLS invasion, and increase the production of matrix metalloproteinases (MMPs), which, in turn, aggravate synovial inflammation resulting in joint destruction [7-11]. FLSs play an essential role as effector cells in joint destruction through the production of MMPs, mainly collagenases and gelatinases [12]. The number of FLSs and inflammatory cells (mainly macrophages) in the joint greatly increases in both the lining and sublining areas of the RA synovium, and they produce various cytokines and MMPs, infiltrate into neighbouring tissues, cause persistent inflammation, and lead to joint destruction [13]. Cartilage destruction is caused by proteolysis induced by MMPs that remodel the extracellular matrix. Furthermore, MMP degrading enzymes remove the extracellular matrix (ECM), providing space for FLS to invade [14]. MMP-2 and MMP-9, also called collagenases, degrade type IV collagen, gelatin and elastin, and are induced in RA-FLS by pro-inflammatory cytokines, through the activation of transcription factors such as nuclear factor-κB (NF-κB) and activator protein-1 (AP-1) [15].

Celastrus belongs to the family *Celastraceae* and is a Chinese herb that has been used for centuries in folk medicine for the treatment of various inflammatory diseases [16]. Plant-derived herbal products are generally less toxic and better tolerated than many conventional drugs in the treatment of RA [17]. Many conventional anti-arthritic drugs are effective in suppressing inflammation but do not offer protection against bone damage [18]. *Celastrus orbiculatus* extract (COE) is purified from the *Celastrus orbiculatus* stem. We previously reported that COE has a variety of anti-tumor effects [19]. Other recent reports suggested that Celastrus extract has beneficial anti-arthritic effects in an adjuvant-induced arthritis (AIA) model [16,20,21]. Studies to define the therapeutic mechanism of Celastrus extract in RA showed that it inhibited inflammation-mediated bone remodelling in an AIA model [22]. However, it’s utility for inhibiting inflammation-induced RA-FLS invasion and the mechanisms involved have not been examined. Therefore, this study aimed to investigate the effects and mechanism of COE on IL-1β and TNF-α combination-stimulated human RA-FLSs migration and invasion.

## Methods

### Plant material and extraction

The stems of Celastrus orbiculatus (batch no. 070510) were obtained from Guangzhou Zhixin Pharmaceutical Co., Ltd. (Guangzhou, China) in 2007, and identified by Professor Qiang Wang, Department of Chinese Materia Medica Analysis, China Pharmaceutical University. A voucher specimen (no. 20071300) was deposited in the same department. Ethanol extract of *Celastrus aculeatus* Merr. (COE) was prepared as previously described [19]. Briefly, stems of Celastrus were dried, powdered and then extracted with 95% ethanol. The final ethyl acetate extract was condensed and finally lyophilized into powder (250 g) and stored at 4°C. The resultant micropowder was diluted in dimethyl sulfoxide (DMSO) to the required concentrations and filtered before use.

### Cell culture

RA-FLSs were isolated and cultured as described previously [23,24]. FLSs were grown in Dulbecco’s modified Eagle’s medium/Nutrient Mixture F-12 (DMEM/F-12) (Gibco, Grand Island, NY, USA) medium containing 10% foetal bovine serum (FBS), supplemented with antibiotics (100 mg/mL streptomycin and 100 U/mL penicillin ) in a humidified incubator at 37°C under 5% CO_2_, 21% O_2_, and 75% N_2_ (Sanyo, Osaka, Japan). Cells used for experiments were at the third to sixth passage. Isolated RA-FLSs were identified by flow cytometry (FCM; BD Biosciences, San Jose, CA, USA) as described previously.

### Cell viability assay

All viability assays were based on the 3-(4, 5-dimethylthiazol-2-yl)-2, 5-diphenyltetrazolium bromide (MTT) method. Briefly, FLSs were seeded in a 96-well plate at a density of 1 × 10^4^ cells/well. After treatment with various concentrations of COE (5, 10, 20, 40 and 80 μg/ml) in triplicate for 20 h, cells were added to wells with 20 μl of MTT (5 mg/ml) per well and incubated for an additional 4 h. Cells were pelleted and lysed in 100 μl of DMSO and the absorbance at 550 nm was measured using a microplate reader (Thermo, Waltham, MA, USA).

### Cell cycle determination

Cell cycle distribution was analysed by FCM. Briefly, FLSs were plated at a density of 1 × 10^6^ cells per 100-mm culture dish and treated with different concentrations (5, 10, 20 and 40 μg/ml) of COE for 24 h. Subsequently, the cells were harvested, washed twice with phosphate buffer saline (PBS), and fixed in 70% ethanol at 4°C for 1 h and centrifuged. Fixed cells were incubated with RNase (50 μg/ml) for 30 min prior to staining nucleic acids with propidium iodide (50 μg/ml) for 30 min at room temperature. The sub G_1_ value in each group was analysed by FCM.

### *In vitro* migration and invasion assay

Cell migration *in vitro* was determined using 6.5 mm Transwell chambers with 8 μm pores (Corning, NY, USA). COE treated-FLSs (1 × 10^5^ cells) were plated in the upper chambers in duplicate filters. In the outer wells, 900 μl DMEM/30% FBS and IL-1β (10 ng/ml), TNF-α (10 ng/ml) or IL-1β (10 ng/ml) and TNF-α (10 ng/ml) (R&D, Minneapolis, MN, USA), were added to the lower chamber. After a 48 h incubation period at 37°C and 5% CO_2_, the cells were fixed with 2% paraformaldehyde in PBS for 30 min at room temperature. After removal of paraformaldehyde and subsequent washing with PBS, the cells were stained with a crystal violet solution for 30 min at room temperature. The non-migrating cells were removed from the upper surface by cotton swabs. Cells that migrated through the membrane to the lower surface were counted in five representative microscopic fields (×100 magnification) and photographed. Cell invasion ability was determined using Matrigel invasion chambers (BD Biosciences, Tokyo, Japan) according to the manufacturer’s instructions. The upper chambers were freshly coated with Matrigel, and medium was added to the lower chamber as described above. RA-FLSs (5 × 10^4^ cells) were suspended in medium containing 2% FBS and seeded into Matrigel pre-coated Transwell chambers. Cell invasion was allowed to occur for 48 h and the gel and cells on the top membrane surface were removed with cotton swabs. Cells that had penetrated to the bottom were counted. All experiments were performed in triplicate and repeated at least twice.

### Quantitative real-time polymerase chain reaction (qRT-PCR)

Total RNA was extracted using Trizol according to the manufacturer’s protocol. A SuperScript™ III Platinum®SYBR® Green one-step qRT-PCR kit (Invitrogen, Carlsbad, CA, USA) was used. Glyceraldehyde 3-phosphate dehydrogenase (GAPDH) was used as the internal control for all analysis. The forward and reverse primers were designed using Primer Express software (version 2.0-PE Applied Biosystems). The sequences of primers used were as follows: MMP-1, 5′- ACT CTG GAG TAA TGT CAC ACC T -3′ (F) and 5′- GTT GGT CCA CCT TTC ATC TTC A -3′ (R); MMP-2, 5′- CCG TCG CCC ATC ATC AAG TT -3′ (F) and 5′- CTG TCT GGG GCA GTC CAA AG -3′ (R); MMP-3, 5′- AGT CTT CCA ATC CTA CTG TTG CT -3′ (F) and 5′- TCC CCG TCA CCT CCA ATC C-3′ (R); MMP-9, 5′- GGG ACG CAG ACA TCG TCA TC -3′ (F) and 5′- TCG TCA TCG TCG AAA TGG GC -3′ (R); GAPDH, 5′- ATC CCG CTA ACA TCA AAT GG-3′ (F) and 5′- GTG GTT CAC ACC CAT CAC AA -3′ (R). Primer specificity was assessed from monophasic dissociation curves, and all had a similar efficiency (data not shown). The threshold cycle (Ct) for the endogenous control GAPDH mRNA and target signals was determined, and relative RNA quantification was calculated using the comparative 2^-ΔΔCt^ method where ΔΔCt = (Ct^Target^ - Ct^GAPDH^) - (Ct^Control^ - Ct^GAPDH^). All reactions were performed in duplicate.

### Enzyme-linked immunosorbent assay (ELISA)

The cell supernatants were collected for measurement of secreted-MMP-1, 2, 3, and 9. Total and active MMP-9 protein was assayed according to the manufacturer’s instructions for MMP-1, 2, 3, and 9 ELISA Systems (GE Healthcare, Tokyo, Japan). MMP-1, 2, 3, and 9 activities were expressed as a change in fluorescence intensity at an excitation wavelength of 490 nm/emission of 520 nm.

### Western blot analysis

After experimental treatment, whole cell lysates from FLSs were generated using a Total Protein Extraction Kit (Millipore, Billerica, MA, USA) according to the manufacturer’s instructions. Protein concentrations were determined using a Pierce BCA Protein Assay Kit (Thermo Scientific, Tokyo, Japan). Equal amounts of protein (30 μg) were separated by 10% sodium dodecyl sulfate-polyacrylamide gel electrophoresis (SDS-PAGE) and transferred to ECL nitrocellulose membranes (Amersham Biosciences, Piscataway, NJ, USA). After blocking with 5% BSA for 2 h, blots were probed with primary antibodies at 4°C for 12 h, including primary antibodies against IκBα (1:400), p65 (1:400), phospho-IκBα (p-IκBα) (1:500), p-p65 (1:500), MMP-2 (1:400), MMP-9 (1:400) and β-actin (1:1000). All primary antibodies were from Santa Cruz Biotechnology (Santa Cruz, CA, USA). Membranes were then incubated with appropriate secondary antibodies for 2 h at room temperature. ECL reagent (GE Healthcare, Tokyo, Japan) was used for protein detection. β-actin was used as an internal control. The relative expression of each protein was determined by densitometric analysis and normalized to the control. Each blot shown is representative of at least three similar independent experiments.

### Gelatin zymography

The enzymatic activities of MMP-2 and MMP-9 were determined by gelatin zymography. Briefly, cells were seeded and allowed to grow to confluence and then incubated in serum-free medium for 24 h. The supernatants were collected 48 h after stimulation, mixed with non-reducing sample buffer, and separated by 10% SDS-PAGE containing 1% gelatin. After electrophoresis, gels were renatured by washing in 2.5% Triton X-100 solution twice for 30 min to remove all SDS. The gels were then incubated in 50 mM Tris–HCl (pH 7.5), 5 mM CaCl_2_, and 1 *μ*M ZnCl_2_ at 37°C overnight. Gels were then stained with 0.25% Coomassie brilliant blue R-250 for 30 min and then destained in distilled water.

### Transient transfection and luciferase reporter assay

To determine promoter activity, we used a dual-luciferase reporter assay system (Promega, Madison, WI, USA). MMP-9 promoter luciferase reporter plasmid and its MMP-9 mutant NF-κB (mNF-κB) and MMP-9 mAP-1 were constructed using standard molecular biology techniques as previously described [25]. RA-FLSs (1 × 10^5^) were seeded into 24-well plates and incubated at 37°C. Cells at 70–80% confluence were co-transfected with reporter constructs and *Renilla* luciferase reporter vector using Lipofectamine 2000 (Invitrogen, Carlsbad, CA, USA) for 24 h according to the manufacturer’s protocol. In the same experiment, we added an empty control plasmid to ensure that each transfection received the same amount of total DNA. The transfected cells were pretreated with COE for 1 h and then stimulated with 10 ng/ml of IL-1β or/and TNF-α for 48 h, respectively. To assess promoter activity, cells were collected and disrupted by sonication in lysis buffer. After centrifugation, aliquots of the supernatants were assayed according to the manufacturer’s protocol (Promega, Madison, WI, USA) using a Luminometer (Turner BioSystems, Sunnyvale, CA, USA). Relative luciferase activity (RLA) was normalized to *Renilla* luciferase activity and expressed as the mean of three independent experiments.

### Electrophoretic mobility shift assay (EMSA)

Cell nuclear lysates were harvested using a NucBuster™ Protein Extraction Kit (Novagen, Germany) according to the manufacturer’s instructions. Nuclear extracts (10 μg) were used to detect NF-κB translocation. Nuclei were resuspended in lysis buffer supplemented with 0.5 mM DTT and 0.2 mM phenylmethylsulfonyl fluoride (PMSF). The NF-κB consensus oligonucleotides (5′-AGT TGA GGG GAC TTT CCC AGG C -3′) labelled with ^32^P by T4 polynucleotide kinase (Promega, Madison, WI, USA) were incubated with nuclear extracts in binding buffer at 30°C for 30 min. The free DNA and DNA-protein mixtures were resolved using a 5% native polyacrylamide gels in 0.5 × TBE buffer (0.4 M Tris, 0.45 M boric acid, 0.5 M EDTA, pH 8.0) by EMSA. Gels were dried and subjected to autoradiography analysis.

### Chromatin immunoprecipitation (ChIP) assay

To detect the *in vivo* association of nuclear proteins with the human MMP-9 promoter, chromatin from FLSs was fixed and immunoprecipitated using the ChIP assay kit as recommended by the manufacturer (Upstate Biotechnology, NY, USA). Immune complexes were prepared using anti-NF-κB p65 antibody. The supernatant of an immunoprecipitation reaction carried out in the absence of antibody was used as the total input DNA control. After DNA purification, the presence of the selected DNA sequence was assessed by PCR. PCR primers for the MMP-9 promoter (373 bp including NF-κB cluster, GenBank accession number AF538844) were as follows: sense (5′-CAC TTC AAA GTG GTA AGA -3′), anti-sense (5′-GAA AGT GAT GGA AGA CTC C -3′). PCR products were resolved by 1.5% agarose gel and visualized with UV light after being stained with ethidium bromide.

### Statistical analysis

All values are expressed as the mean ± SD, unless otherwise stated. Results from different groups were analysed by one-way analysis of variance (ANOVA) with Fisher’s probable least-squares difference test or Student’s *t*-test. Statistical analysis was performed using SAS 9.2 software (SAS Institute Inc., NC, USA). Differences resulting in probability (*P*) values less than 0.05 were considered statistically significant.

## Results

### Effect of COE on IL-1β and TNF-α-induced FLSs migration and invasion

**Figure 1:**
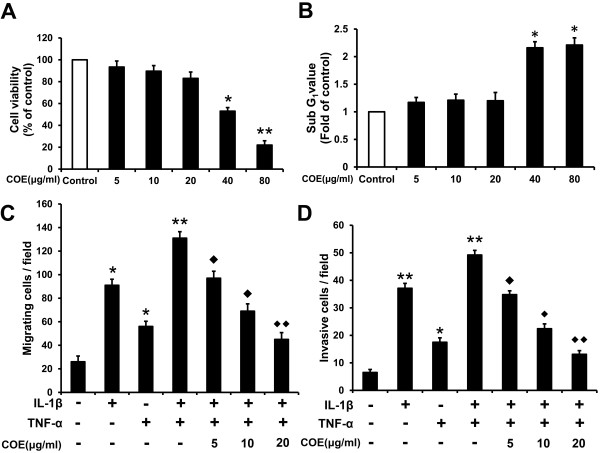
**Effect of COE on IL-1β and TNF-α-induced FLSs migration and invasion. (A)** FLSs were incubated with the indicated concentrations of COE in serum containing medium for 24 h, and cell viability was measured by MTT assay. ^*^P < 0.05, ^**^P < 0.01 versus normal control group. **(B)** FLSs were incubated with the indicated concentrations of COE for 24 h. Cells were harvested and the cell cycle distribution in the sub-G1 phase was determined by FCM analysis. ^*^P < 0.05 versus normal control group. The migration **(C)** and invasion **(D)** abilities of FLSs were determined by cell migration and invasion assays. FLSs were pretreated with the indicated concentrations of COE for 1 h. Then, FLSs were allowed to migrate with or without IL-1β (10 ng/ml), TNF-α (10 ng/ml), or IL-1β (10 ng/ml) and TNF-α (10 ng/ml) for 48 h, respectively. The number of migrating and invasive cells in each chamber was plotted as the mean ± SD in three independent experiments. The results were analysed by ANOVA. ^*^P < 0.05, ^**^P < 0.01 versus normal control group, ^◆^*P* < 0.05, ^◆◆^*P* < 0.01 versus IL-1β and TNF-α combination-treated group.

As shown in Figure 1(A), this experiment was undertaken to examine the cytotoxic effect of COE on human RA-FLSs. FLSs were treated with various concentrations of COE in serum-containing medium for 24 h, and cell viability was determined using the MTT assay. Treatment with 5 to 20 μg/ml COE had no significant effect on cell viability at 24 h. However, 40 μg/ml and 80 μg/ml COE decreased cell viability by approximately 1 and 2.5-fold, respectively, in comparison with the control group. As shown in Figure 1(B), cell cycle analysis by FCM showed that COE did not influence the cell cycle transition at low doses (5, 10 and 20 μg/ml), whereas high doses (40 and 80 μg/ml) caused sub-G1 accumulation. Therefore, COE had no significant cytotoxicity in FLSs at low doses. Based on data from preliminary studies, three different concentrations of COE (5, 10 and 20 μg/ml) were chosen for the following experiments. Whether COE could inhibit IL-1β and TNF-α-induced cell migration and invasion was analysed using a Transwell chamber. As shown in Figure 1(C) and (D), FLS migration was significantly induced by IL-1β from a control level of 26 cells/field to 91 cells/field (*P* < 0.05). This effect was similar to that of TNF-α and their combination was more active, inducing migration of 131 cells/field. Similarly, data obtained from the invasion assay showed that combined IL-1β and TNF-α increased cell invasion from a control level of 6.5 cells/field to 49.2 cells/field (*P* < 0.01). However, IL-1β and TNF-α combination-induced cell migration and invasion were inhibited by COE in a dose-dependent manner. These results suggest that non-toxic concentrations of COE ranging from 5 to 20 μg/ml could inhibit RA-FLSs migration and invasion induced by IL-1β and TNF-α *in vitro*, indicating COE might provide benefit in the treatment of RA by inhibiting inflammation-induced FLSs invasion.

### Effect of COE on mRNA expression of MMPs in IL-1β and TNF-α-induced FLSs

**Figure 2:**
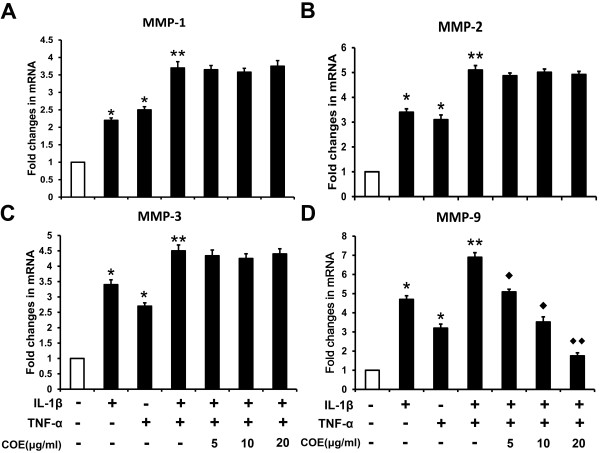
**Effect of COE on mRNA expression of MMPs in IL-1β and TNF-α-induced FLSs.** FLSs were incubated with the indicated concentrations of COE for 1 h followed by IL-1β (10 ng/ml) and TNF-α (10 ng/ml) stimulation. After 48 h, the mRNA levels of endogenous MMP-1**(A)**, MMP-2 **(B)**, MMP-3 **(C)** and MMP-9 **(D)** were measured by qRT-PCR. GAPDH and β-actin were used as internal controls, respectively. The histogram shows the mRNA levels from three independent experiments. ^*^P < 0.05, ^**^P < 0.01 versus normal control group, ^◆^*P* < 0.05, ^◆◆^*P* < 0.01 versus IL-1β and TNF-α combination-treated group.

As COE inhibited FLSs migration and invasion, this prompted us to examine the effect of COE on MMP gene expression. After stimulation of FLS with IL-1β (10 ng/ml), TNF-α (10 ng/ml) or IL-1β (10 ng/ml) and TNF-α (10 ng/ml), MMP mRNA expression was determined. Figure 2 shows the mean levels for MMP-1, -2, -3 and -9 mRNA expression induced by two different cytokines after 48 h of stimulation and pretreatment with COE. mRNA expression of MMP-1 was similar with MMP-2 after stimulation with IL-1β or TNF-α for 48 h, although TNF-α induced a lower mRNA expression of MMP-3 and MMP-9 compared with IL-1β. Our results showed that combined IL-1β and TNF-α induced MMP-1, -2, -3 and -9 mRNA expression was higher than IL-1β or TNF-α alone. There was a synergistic effect in the IL-1β and TNF-α induced MMP-1, -2, -3 and -9 mRNA expression, whereas treatment with COE suppressed IL-1β and TNF-α-induced MMP-9 expression in a dose-dependent manner. Significant inhibition was seen for MMP-9 mRNA expression after 48 h incubation with IL-1β and TNF-α (5 μg/ml or higher COE). However, MMP-1, -2 and -3 mRNA expression was not affected by COE pretreatment.

### Effect of COE on MMP-2 and MMP-9 activity in IL-1β and TNF-α-induced FLSs

**Figure 3:**
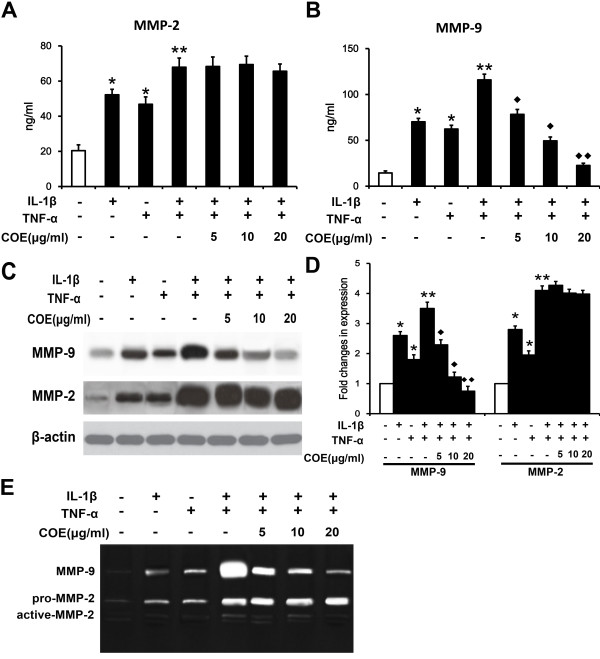
**Effect of COE on MMP-2 and MMP-9 activity in IL-1β and TNF-α-induced FLSs.** FLSs were pretreated with the indicated concentrations of COE for 1 h followed by IL-1β (10 ng/ml) and TNF-α (10 ng/ml) stimulation. After 48 h, the supernatants were collected and assayed for the amount and activity of secreted MMP-2 and MMP-9, by ELISA **(A and B)**, western blot **(C and D)** and gelatin zymography **(E)** respectively. The histogram shows the ELISA and western blot results from three independent experiments. ^*^P < 0.05, ^**^P < 0.01 versus normal control group, ^◆^*P* < 0.05, ^◆◆^*P* < 0.01 versus IL-1β and TNF-α combination-treated group.

Figure 3 shows the production of MMP-2 and MMP-9 by FLSs after stimulation with IL-1β (10 ng/ml), TNF-α (10 ng/ml) or IL-1β (10 ng/ml) and TNF-α (10 ng/ml), and also after pretreatment with COE (5 μg/ml, 10 μg/ml or 20 μg/ml). As shown in Figure 3(C) and (D), there was an obvious synergistic effect for the IL-1β and TNF-α induced MMP-9 and MMP-2 protein expression, whereas treatment with COE suppressed MMP-9 expression in a dose-dependent manner. However, MMP-2 protein expression was not affected by COE treatment. We next examined the effect of COE on the secretion and proteolytic activity of MMP-2 and MMP-9 protein levels in conditioned medium. As shown in Figure 3(A-B) and (E), the secretion and proteolytic activity of MMP-9 in FLSs were induced when FLSs were cultured in serum-free medium with 10 ng/ml of IL-1β or/and TNF-α for 48 h. The treatment of FLSs with COE suppressed IL-1β and TNF-α-induced MMP-9 secretion and activity in a dose-dependent manner. These results indicate that COE selectively inhibits IL-1β and TNF-α-induced MMP-9 expression at both the gene and protein levels, which subsequently suppresses the enzymatic activity of MMP-9.

### COE inhibits the transcriptional activity of MMP-9 by suppression of NF-κB activity

**Figure 4:**
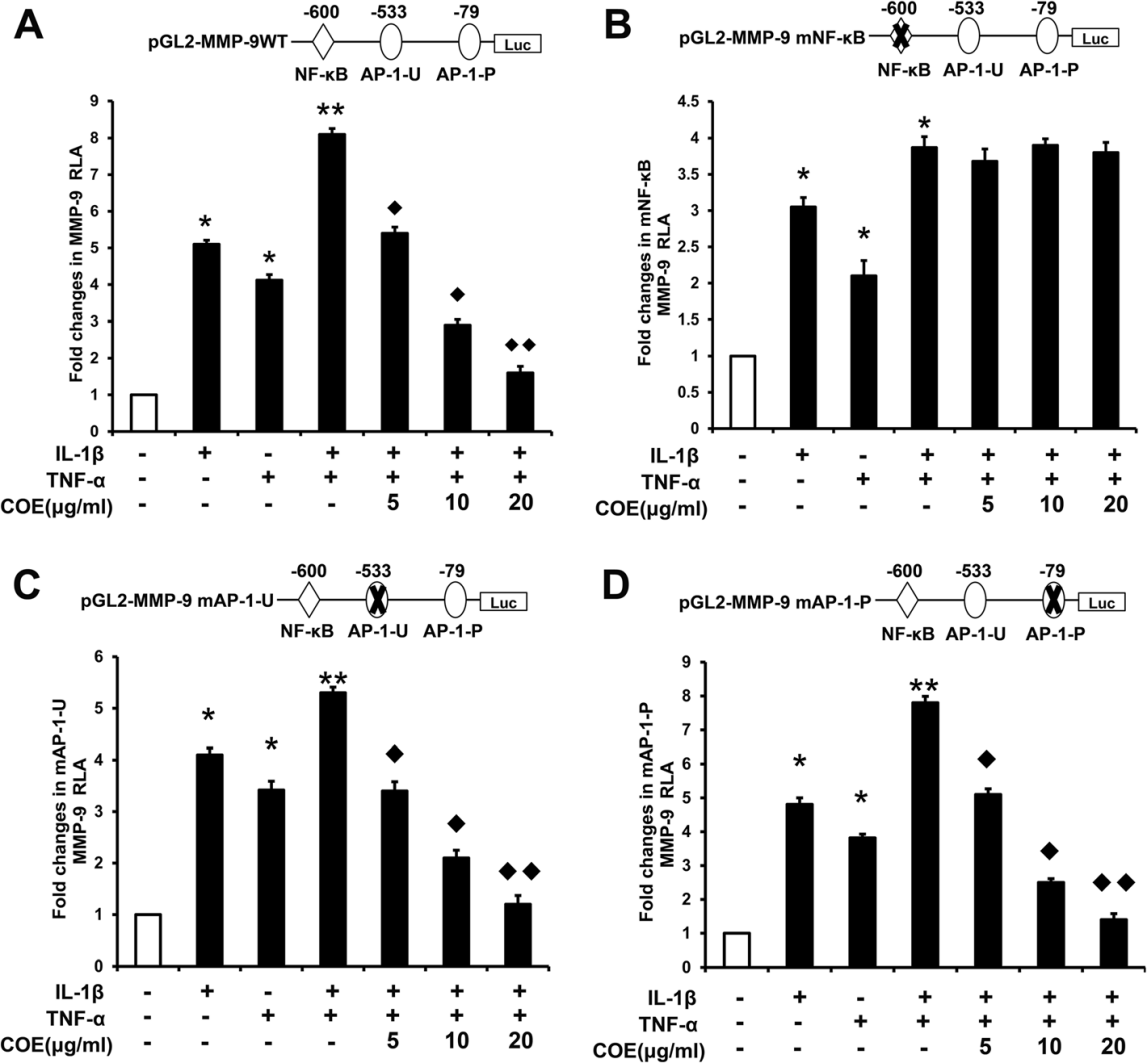
**COE inhibits the transcriptional activity of MMP-9 by suppression of NF-κB activity. (A)** RA-FLSs were transfected with pGL2-MMP-9WT and *Renilla* luciferase reporter vector plasmids. The transfected cells were pretreated with the indicated concentrations of COE for 1 h, followed by IL-1β (10 ng/ml) and TNF-α (10 ng/ml) stimulation for 48 h. The relative luciferase activity in the cell extract was normalized by *Renilla* luciferase activity. Each value represents the mean ± SD of triplicate experiments and is expressed relative to the control. FLSs were transfected with pGL2-MMP-9 mNF-κB **(B)**, pGL2-MMP-9 mAP-1-U **(C)** and pGL2-MMP-9 mAP-1-P **(D)**. The relative luciferase activity is normalized to *Renilla* luciferase activity and is expressed relative to the controls. ^*^P < 0.05, ^**^P < 0.01 versus normal control group, ^◆^*P* < 0.05, ^◆◆^*P* < 0.01 versus IL-1β and TNF-α combination-treated group.

**Figure 5:**
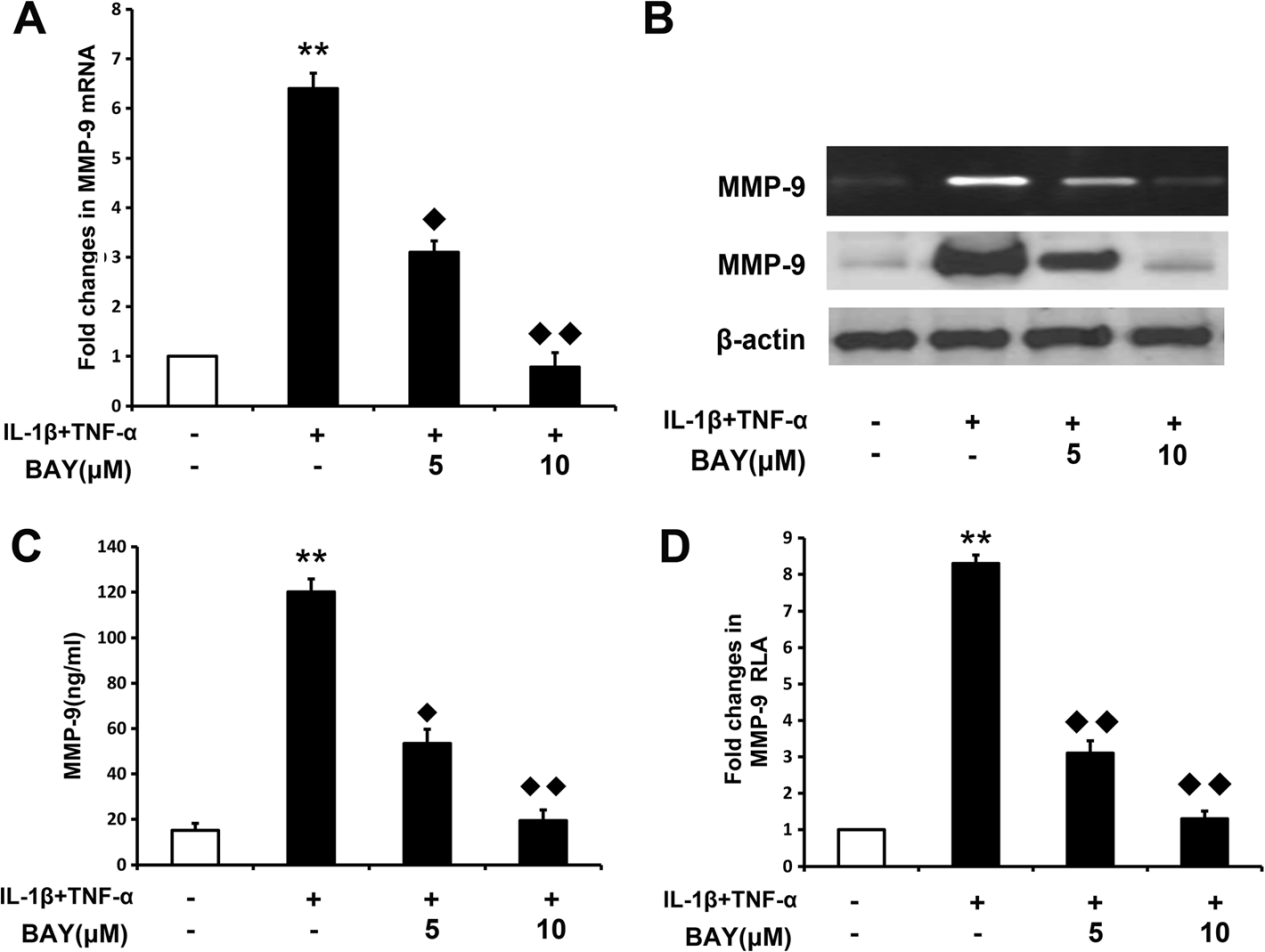
**NF-κB mediates IL-1β and TNF-α-induced MMP-9 expression. (A)** FLSs were pretreated with BAY (5, 10 μM) for 1 h and then stimulated with IL-1β (10 ng/ml) and TNF-α (10 ng/ml) for 48 h. MMP-9 mRNA expression in cells was analysed by qRT-PCR. GAPDH was used as an internal control. The histogram shows the mRNA levels from three independent experiments. **(B)** and **(C)** FLSs were pretreated with BAY (5, 10 μM) for 1 h and then stimulated with IL-1β (10 ng/ml) and TNF-α (10 ng/ml). After 48 h, conditioned media was collected and gelatin zymography, western blotting or ELISA was performed. **(D)** FLSs were transfected with pGL2-MMP-9WT reporter plasmids and then cultured with BAY (5, 10 μM) with or without IL-1β (10 ng/ml) and TNF-α (10 ng/ml) for 48 h. Luciferase activity in the cell extracts was determined. The data are presented as mean ± SD of triplicate experiments. ^**^P < 0.01 versus normal control group, ^◆^*P <* 0.05, ^◆◆^*P <* 0.01 versus IL-1β and TNF-α combination-treated group.

The two principal pathways activated by IL-1 and TNF-α are the NF-κB and AP-1 pathways [10,18], which induce MMP-9 expression. The MMP-9 gene is regulated at the transcriptional level by interactions of NF-κB and AP-1 with their binding sequences in the MMP-9 promoter [26]. Because COE suppresses MMP-9 mRNA expression, we tested which of these transcription factors might regulate the MMP-9 gene in FLSs. FLSs were transiently transfected with reporter genes that included the wild-type MMP-9 promoter or a promoter with mutations in the NF-κB site or in one or both AP-1 sites. As shown in Figure 4, treatment with COE in the presence of IL-1β and TNF-α decreased the transcriptional activity of the reporter with the AP-1 mutation, but had no effect on the reporter with NF-κB mutations, suggesting that the target of COE is the NF-κB transcription factor. Furthermore, the NF-κB inhibitor BAY, which blocks the nuclear translocation of NF-κB, was used to examine the involvement of NF-κB in IL-1β and TNF-α-induced MMP-9 activation. FLSs were pretreated with BAY (5 and 10 μM) for 1 h and then stimulated with 10 ng/ml of IL-1β and TNF-α for 48 h. qRT-PCR showed that treatment of FLSs with BAY decreased IL-1β and TNF-α-stimulated MMP-9 mRNA expression (Figure 5A), indicating that the NF-κB inhibitor prevented the induced transcription of MMP-9. Culture media were subjected to gelatin zymography and western blot analysis. As shown in Figure 5(B) and (C), BAY inhibited IL-1β and TNF-α-induced MMP-9 protein expression and activation. The effect of the NF-κB inhibitor on MMP-9 promoter activity was investigated using FLSs transiently transfected with a luciferase reporter gene linked to the MMP-9 or NF-κB promoter sequence. As shown in Figure 5(D), treatment of FLSs with the NF-κB inhibitor clearly decreased the IL-1β and TNF-α-induced luciferase activity.

### COE inhibits the binding activity of NF-κB in the MMP-9 promoter

**Figure 6:**
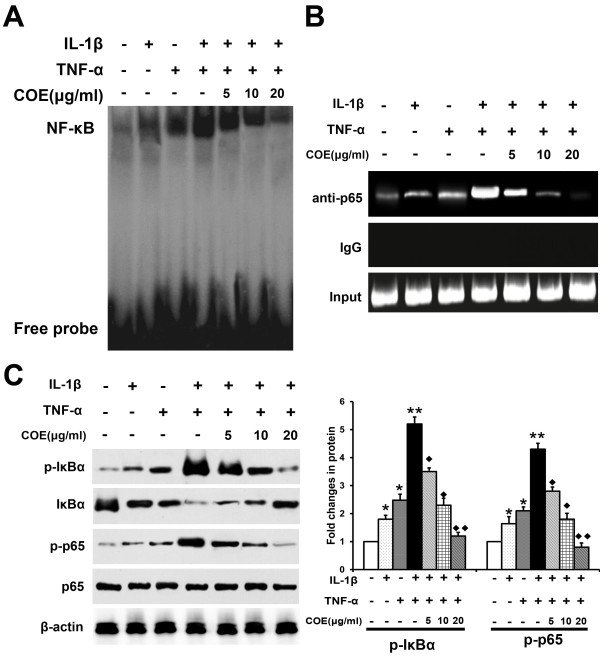
**COE inhibits the binding activity of NF-κB in the MMP-9 promoter.** FLSs were pretreated with the indicated amounts of COE for 1 h, and stimulated with IL-1β (10 ng/ml) and TNF-α (10 ng/ml) for 48 h. **(A)** The DNA binding activity of the nuclear extracts was examined by EMSA using a probe containing the NF-κB motif in the MMP-9 promoter. NF-κB DNA binding activity was analysed by EMSA and shown is a representative blot from three independent experiments. **(B)** The cross-linked chromatin was prepared and immunoprecipitated with antibody and IgG to NF-κB p65 before amplification of the MMP-9 gene region containing the NF-κB site. Immunoprecipitates were analysed by PCR for the presence of the MMP-9 gene promoter sequence using the primer pair described in material and methods. DNA purified from the sonicated chromatin was directly analysed by PCR using the same primer set, which served as an input control (Input). Similar results were obtained in three independent experiments. **(C)** FLSs were harvested and whole cell extracts were subjected to western blot analysis for the indicated proteins (IκBα, p-IκBα, p65 and p-p65). A representative protein blot of three independent experiments is shown. β-actin served as a control. The histogram shows the protein relative expression changes from three independent experiments. ^*^P < 0.05, ^**^P < 0.01 versus normal control group, ^◆^*P* < 0.05, ^◆◆^*P* < 0.01 versus IL-1β and TNF-α combination-treated group.

We examined the inhibitory effect of COE on the binding of NF-κB isolated from IL-1β and TNF-α-stimulated FLSs to oligonucleotides that contained NF-κB binding sites in the MMP-9 promoter using EMSA. As shown in Figure 6(A), *in vitro* COE treatment suppressed IL-1β and TNF-α-induced NF-κB binding to the MMP-9 promoter on EMSA. Furthermore, we used ChIP to determine whether COE-suppressed NF-κB complexes could bind with the MMP-9 promoter *in vivo*. Chromatin was extracted and immunoprecipitated using anti-NF-κB antibody, and the MMP-9 promoter region was amplified by PCR. As shown in Figure 6(B), *in vivo* binding of NF-κB to the MMP-9 promoter increased in response to IL-1β and TNF-α treatment, whereas NF-κB binding to the MMP-9 promoter was significantly inhibited by COE. These results further demonstrate that COE suppresses IL-1β-induced MMP-9 transcription through the control of NF-κB. To characterize the molecules involved in the inhibitory effect of COE, we next examined whether COE regulated the NF-κB signalling pathway. FLSs were pretreated with different concentrations of COE for 1 h and stimulated with IL-1β and TNF-α for 48 h and then levels of the total or phosphorylated forms of IκBα and p65 were measured by western blot. As shown in Figure 6(C), IL-1β or/and TNF-α stimulation induced significant phosphorylation of IκBα, and IκBα degradation, but also increased phosphorylation of p65. COE markedly inhibited the IL-1β and TNF-α-induced phosphorylation of IκBα and p65 in a dose-dependent manner. Increased IκBα levels correlated with a constant decrease of phosphorylated IκBα in FLSs. These results suggest that COE inhibits IL-1β and TNF-α-induced NF-κB activation by suppressing IκBα phosphorylation and the nuclear translocation of NF-κB in RA-FLSs. Thus, COE inhibits NF-κB-dependent transcriptional activation, resulting in the suppression of IL-1β and TNF-α-induced MMP-9 expression in RA-FLSs.

## Discussion

Celastrus has been utilized as a medicinal herb in traditional Chinese medicine for the treatment of arthritis for many decades [27]. Although several studies have shown that Celastrus has anti-arthritic activities [21,27], the precise mechanisms by which it can alleviate the clinical symptoms of RA patients are not well defined. It was confirmed that human RA-FLSs express oncogenes that are characteristic of actively dividing cells. Hence, the growth and motility of FLSs from RA patients is uncontrolled, resulting in excessive proliferation and invasion. Therefore, tumor therapy might be useful for RA treatment. Our previous studies demonstrated that COE has a variety of anti-tumor effects. Therefore, the present study was undertaken to examine the possible therapeutic mechanisms of Celastrus on RA-FLSs migration and invasion *in vitro.* In this study, we assessed the effect of COE on IL-1β and TNF-α-induced RA-FLSs motility. Our results clearly showed that treatment of RA-FLSs with COE suppressed IL-1β and TNF-α-induced cell migration and invasion, and revealed that COE inhibited the transcriptional activity of MMP-9 by suppressing the binding activity of NF-κB in the MMP-9 promoter, and inhibited IκBα phosphorylation and NF-κB nuclear translocation.

MMP-2 and MMP-9 are important ECM-degrading enzymes, and overexpression of MMPs is important for the invasiveness of RA-FLSs [28,29]. IL-1β and TNF-α are important pro-inflammatory cytokines in the RA-FLS microenvironment that stimulate FLS to secrete MMPs. This induction is regulated at the transcriptional and translational levels [14]. In the present study, IL-1β and TNF-α induced MMP-1, -2, -3 and -9 expression with an obvious synergistic effect. Furthermore, increased MMP-9 expression and secretion was inhibited by COE. These results therefore indicate that the inhibition of IL-1β and TNF-α-induced FLSs invasion by COE occurs primarily by inhibiting MMP-9 expression and activity. The two principal pathways activated by IL-1β and TNF-α are the NF-κB and mitogen-activated protein kinase (MAPK) pathways, and the roles of both in the pathogenesis of destructive arthritis have been reported [9,10]. The MMP-9 promoter region contains a *cis*-regulatory element, including one NF-κB, two AP-1 and one stimulatory protein-1 (SP-1) binding sites [30]. To identify the mechanism of COE-induced inhibition of MMP-9 expression, we examined MMP-9 promoter activity using wild type and mutant reporter plasmids. COE suppressed MMP-9-induction by repressing transcription activation of the MMP-9 promoter. Mutational analysis of the promoter revealed that the major target of COE was NF-κB, which was further confirmed by the use of reporter plasmids containing synthetic elements specific for the transcription factors.

Next, we investigated the functional significance of NF-κB transactivation of MMP-9 activation in RA-FLSs. Results from *in vitro* EMSA and *in vivo* ChIP assays showed that COE suppressed IL-1β and TNF-α-induced NF-κB binding to the MMP-9 promoter. Given that NF-κB regulates transcriptional activation of multiple inflammatory cytokines, we expected that COE might target NF-κB to suppress MMP-9 transcription by IL-1β and TNF-α. NF-κB is sequestered in the cytoplasm by binding to IκB family molecules and is activated by IκBα phosphorylation whose subsequent degradation in the proteasome allows the NF-κB subunits, p65 and p50, to enter the nucleus and activate target genes [31]. To address whether COE modulated the NF-κB signalling pathway, we attempted to analyze the presence of native and phosphorylated forms of IκBα in the absence or presence of COE. We showed that IL-1β and TNF-α induced phosphorylation of IκBα and triggered degradation of IκBα in RA-FLSs with a synergistic effect and that IL-1β and TNF-α inhibited the effect in a dose-dependent manner. Phosphorylation of p65 by IL-1β is associated with nuclear translocation and transactivation potential [32]. We also showed that COE inhibited the IL-1β and TNF-α-induced phosphorylation of p65.

**Figure 7:**
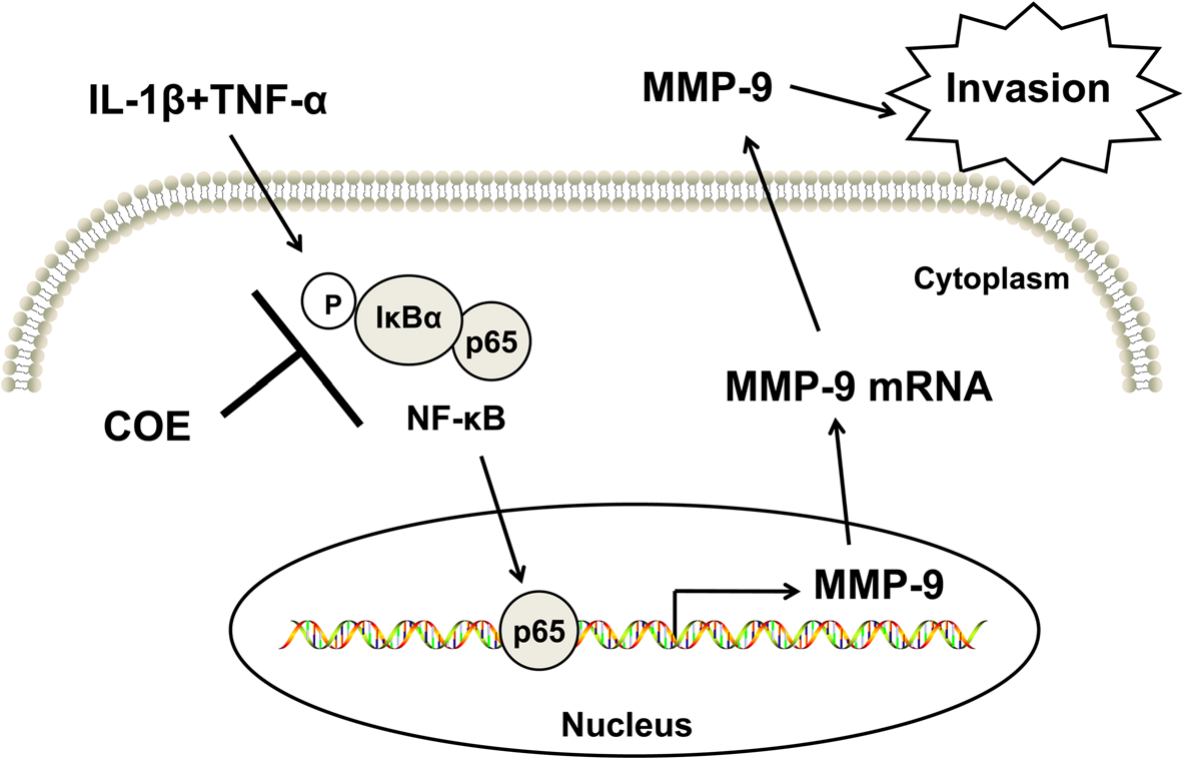
**Schematic diagram.** COE inhibits the IL-1β and TNF-α-induced migration and invasion signal pathway in FLSs. COE blocks IL-1β and TNF-α-induced phosphorylation and degradation of IκBα, which inhibits the nuclear translocation of p65. This further suppresses the activation of NF-κB, and, thus, decreases MMP-9 expression and the subsequent migration and invasion of FLSs.

The above findings collectively demonstrate that COE inhibits IL-1β and TNF-α-induced NF-κB activity via the IκB pathway, inhibits MMP-9 expression and proteolytic activity, which in turn, suppresses the migration and invasion ability of FLSs (Figure 7). Several conventional drugs are available for treating RA, however, the effect of different therapeutic agents on inflammation and bone damage may be separate. Certain drugs can suppress inflammation effectively, but fail to protect against bone erosion, whereas under other conditions, bone erosion can be halted, but inflammation may continue unabated. In this context, our results show that COE may control both inflammation and bone damage in RA pathogenesis.

## Conclusion

Taken together, the present study indicates that COE inhibits IL-1β and TNF-α-induced RA-FLSs migration and invasion by suppressing NF-κB-mediated MMP-9 expression. Although further work is needed to clarify the active ingredients and complicated mechanism of COE-induced anti-invasion effect on FLSs, we suggest that COE is a promising agent for the concurrent treatment of inflammation and bone damage associated with arthritis. Furthermore, natural products should be further tested in clinical studies for their use as adjuncts to conventional drugs for the treatment of RA.

## Competing interests

There is no declared conflict of interests in this study.

## Authors’ contributions

GQL designed the research, performed the experiments, analysed data and wrote the paper; DL performed the experiments; SYG, MS and TH provided technical support and all the reagent and chemical; YQL collected the plant and carried out the extraction. All authors read and approved the final manuscript.

## Pre-publication history

The pre-publication history for this paper can be accessed here:

http://www.biomedcentral.com/1472-6882/14/62/prepub
